# Interaction of sensory processing and balance in adult cochlear implant users

**DOI:** 10.1007/s00405-024-08776-w

**Published:** 2024-07-08

**Authors:** Sevgi Kutlu, Zehra Aydogan, Banu Baş, Cem Meço, Suna Tokgoz-Yilmaz

**Affiliations:** 1 Ankara University Faculty of Health Sciences, Department of Audiology, Ankara, Turkey; 2 Department of Otorhinolaryngology, Ankara University, Faculty of Medicine, Ankara, Turkey; 3 Ankara Yıldırım Beyazıt University, Diseases İbn-i Sina Hospital Altındağ Faculty of Health Sciences, Department of Audiology, Ankara, Turkey

**Keywords:** Sensory integrity, Cochlear implant, Balance, Hearing loss, Quality of life

## Abstract

**Puropse:**

The aim of this study was to evaluate the sensory processing skills, quality of life and balance performance in adult cochlear implant users.

**Methods:**

A sample of 40 individuals was studied in two groups; 20 normal hearing individuals (37.84 ± 15.39 years old) and 20 cochlear implant users (35.58 ± 11.22 year old). Balance performance was assessed by Computerized Dynamic Posturography. The Adolescent/Adult Sensory Profile completed by the individual was used to assess sensory processing skills. Finally, quality of life was assessed with the Short Form-36.

**Results:**

Among the different sensory processing parameters in the adolescent/adult sensory profile, a significant difference was found between the two groups in the parameters of low registration, sensory sensitivity and sensory avoidance (*p* < .05). Visual, vestibular and composite scores were found to be lower in cochlear implant users by Computerised Dynamic Posturography assessment (*p* < .05). When assessing quality of life, emotional well-being and social functioning parameters were found to be lower in cochlear implant users (*p* < .05).

**Conclusion:**

Factors such as the inability to reach sufficient auditory stimuli due to hearing loss and the occurrence of vestibular problems after cochlear implant surgery limit the quantity and quality of sensory stimuli from the environment. Individuals with cochlear implants may prefer to live isolated from society because they cannot adequately process incoming sensory stimuli due to hearing/balance problems, and this may negatively affect the quality of life of individuals. Our findings revealed the necessity of multisensory assessment and therapy protocols when rehabilitating individuals with cochlear implants.

## Introduction

Sensory integrity is concerned with the nervous system’s ability to receive, interpret, organize and respond to incoming sensory stimuli [1]. Sensory integrity organizes the information recognized by the senses of sight, hearing, smell, taste, touch, movement, gravity and posture. The senses allow us to experience and respond to our environment and when the senses work properly and in harmony with each other, adaptation to the environment increases [2, 3]. Disruption of one or more sensory inputs prevents the correct processing of this sensory information in daily life [4] All information from the environment is received through sensory receptors. Vestibular sensation provides information about the position of our head relative to the earth’s surface, the movement of our body in space and balance, while proprioceptive sensation provides information about body position and the movement of our body parts. Visual and auditory senses, on the other hand, provide information about images and sounds coming from the environment without any actual contact with our eyes and ears [3]. Pathologies affecting the visual, vestibular and somatosensory systems, which are parts of sensory processing, result in balance disorders [5].

Cochlear implant is a hearing prosthesis that is surgically implanted in the inner ear and electrically stimulates the auditory nerve fibers of patients with severe/profound sensorineural hearing loss who cannot benefit from conventional hearing aids [6]. Dizziness is a frequently reported complication after cochlear implant surgery [7]. Cochlear implantation has been shown to reduce postoperative vestibular function in the implanted ear [8]. Trauma to the inner ear during cochlear implant surgery poses an inherent risk that can lead to postoperative dizziness and imbalance. The function of the labyrinth may be affected by vestibular damage due to electrode placement or current propagation through the electrode array [9]. Vestibular disturbances after cochlear implant surgery are transient in most cases. However, resolution of symptoms may take weeks to months in some cases and permanent dysfunction may occur in others [10, 11]. Factors such as the inability to reach sufficient auditory stimuli due to hearing loss and the occurrence of vestibular problems that may be seen after cochlear implant surgery may limit the amount and quality of sensory stimuli that individuals with hearing loss obtain from their environment. Due to problems such as the processing of incoming sensory stimuli, they may prefer to live isolated from the society and this may negatively affect the quality of life of individuals.

The aim of this study was to evaluate the sensory processing skills, quality of life and balance performance in adult cochlear implant users.

## Materials and methods

Ankara University Faculty of Medicine Human Research Ethics Committee approved this study with decision number and code İ01-21-23. Informed consent was obtained from all participants, indicating their willingness to participate in the study.

### Study participants

The study included 20 adult implant users with bilateral severe to profound sensorineural hearing loss who had been using unilateral cochlear implants regularly for at least six months. They had no inner ear abnormalities and no physical disability that would cause balance problems, and 20 age- and sex-matched normal hearing subjects.

### Research protocol

After cochlear implant users and individuals with normal hearing completed a demographic information form and provided a general health history, all participants underwent a hearing assessment. Vestibular assessments were then performed using Computerised Dynamic Posturography. Finally, all participants were asked to complete the Short Form-36 (SF-36) and the Adolescent/Adult Sensory Profile.

### Assessment tools

### Pure tone audiometric testing

Pure tone hearing was assessed in a quiet room using TDH-39 headphones and a GSI AUDIOSTAR PRO (Minnesota, USA) audiometer with air conduction thresholds at frequencies of 125–8000 Hz. The pure-tone average was determined by averaging four frequency bands (at 0.5, 1, 2, and 4 kHz) for the right and left ears, respectively. A pure tone mean < 25 dB was considered normal hearing [12]. Hearing aid thresholds of the cochlear implant group were assessed in the free field.

### Computerised dynamic posturography (CDP)

Posturographic testing was performed using the Equitest posturograph manufactured by NeuroCom International®. Computerised dynamic posturography, a valuable tool for investigating sensory, motor and central adaptive disorders, provides an objective measure of postural control. The test apparatus consists of a movable platform on which the patient stands and a movable cabinet surrounding the platform. The Sensory Organisation Test in Computerised Dynamic Posturography objectively identifies abnormalities using the three sensory systems that contribute to postural control: somatosensory, visual and vestibular. The test is performed using six sensory stimulation conditions in which the visual stimuli are varied and the foot support platform is rotated or the visual environment is moved. By deactivating certain elements of the balance system and analysing the results, it is possible to determine which component of the balance system is weak. During the sensory organisation test, the Composite Balance Score (COMP), which is the weighted average of 6 separate scores, assesses the degree to which the patient relies on somatosensory (SOM), visual (VIS) and vestibular (VES) inputs to maintain balance and the degree to which the patient relies on visual information (PREF), whether accurate or not [13–15].

### Short Form-36 (SF-36)

Consisting of 36 items, it is a tool to assess the health status of the person with 8 sub-parameters (physical function, body pain, limitation due to physical problems, emotional well-being, social function, energy/fatigue, general health perception) [16].

### Adolescent/Adult sensory Profile

The Adolescent/Adult Sensory Profile is a standardised 60-item test that has been used since 2002 to assess response to different sensory stimuli (taste/smell, movement, vision, touch, auditory development, activity level). It is used for adolescents/adults aged 11 and over. It can be scored according to norms established for 3 different age groups: 11–18 years, 18–65 years and over 65 years. It consists of four subscales based on Dunn’s theory of sensory processing. In each scale, people are rated as much more than most people, much more than most people, similar to most people, less than most people and less than most people.

1. Sensory sensitivity: Having a low threshold for sensory stimuli and responding to them more than normal.
2. Sensation avoiding: Deliberately avoiding sensory stimuli.
3. Low registration: Responding less or slower than normal to sensory input.
4. Sensation seeking: Enjoying sensory inputs, being in sensory seeking [17].

### Statistical analysis

The data were analysed using the Statistical Package for the Social Sciences (SPSS) 22.0. The data were first analysed for conformity to normal distribution (histogram, Kolmogorov-Smirnov test, QQ-plot). Descriptive statistics were presented as number, percentage, mean ± standard deviation and median (minimum-maximum). Mann-Whitney U and Kruskall-Wallis tests, which are non-parametric tests, were used when variables did not follow a normal distribution, and Student’s t-test and one-way ANOVA tests, which are parametric tests, were used when variables followed a normal distribution. SF-36, CDP and sensory profile scores were compared between the two groups.

## Results

A total of 40 individuals, 20 in the control group and 20 in the cochlear implant (CI) user group, participated in the study. The mean age of the cochlear implant users was 37.84 ± 15.39 years. CI user group’s hearing thresholds were bilaterally severe to profound and all were unilateral cochlear implant users. The mean duration of cochlear implant use was 34.45 ± 31.30 months (min 8 months; max 108 months). All cochlear implant users had free field thresholds between 20 and 40 dB with implants. The mean age of the control group was 35.58 ± 11.22 years. Hearing thresholds were better than 25 dB HL at all frequencies, (right pure tone average 6.26 ± 2.37 dB HL, left püre tone average 4.70 ± 3.38 dB HL).

None of the subjects in either group had a diagnosis (Meniere’s disease, BPPV, etc.) that would cause vestibular problems. However, 7 of the cochlear implant users had complaints of imbalance that started after cochlear implant surgery.

Descriptive characteristics of the groups was given in Table 1 and the groups were similar in terms of gender (*p* = .125) and age (*p* = .092).

**Table 1:** Comparison of Groups According to Their Descriptive Characteristics

		Control Group (*n* = 20)	CI Group (*n* = 20)	*p*
Age (year)	Mean ± SD (Min-Max)	35.58 ± 11.22 (21–58)	37.84 ± 15.39 (18–64)	0,092
Gender	Female (n) Male (n)	11 9	10 10	0,125

When the SF-36 Quality of Life scores of the control group and the CI group were compared, a significant difference was obtained in the E and SF sub-parameters. E (*p* = .016) and SF (*p* = .022) scores of the CI group were worse than the control group (Table 2).

**Table 2:** Comparison of SF-36 between groups

Control Group		CI Group		%95CI CI [lower/upper]
SF-36	**X ± SS**	**X ± SS**	**p**	
PF	92.89 ± 12,83	85.53 ± 16,98	0.140	[-2.53/17.27]
PR	72.37 ± 36,22	76.32 ± 36.77	0.741	[-27.96/20.07]
EP	80.70 ± 32.04	75.44 ± 34.85	631	[-16.76/27.29]
E/F	53.16 ± 18.94	55.26 ± 30.43	0.799	[-18.78/14.57]
E	64.84 ± 15.75	54.63 ± 23.67	0.016*	**[-5.08/21.44]**
SF	71.68 ± 18.99	65.79 ± 25.29	0.022*	**[-8.82/20.61]**
P	71,09 ± 10,75	75.39 ± 24.22	0.161	[-4.61/26.72]
GH	67.42 ± 17.74	61.58 ± 22.11	0.375	-7.349/19.03]

Data were presented as mean ± standard deviation and CI (confidance interval). The SF-36 consists of 36 items – that are grouped in 8 subscales or domains; PF: Physical functioning, PR: Role limitations due to physical health, EP: Role limitations due to emotional problems, E/F: Energy/fatigue, E: Emotional well-being, SF: Social functioning, P: Pain, GH: General health.Within- group changes were presented as * *p* < .05 and ** *p* < .01. X ± SD: Mean ± Standart Deviation, Paired samples T Test *p < 0,05, Wilcoxon signed rank test *p < 0,05**p < 0,001

When the sensory scores of the control group and the CI group were compared, significant differences were obtained in the scores of low registration (*p* = .001), sensory sensitivity (*p* = .014), sensory avoidance (*p* = .035), motor processing (*p* = .021), tactile processing (*p* = .002), activity level (*p* = .006) and auditory processing (*p* = .024). The CI group had worse sensory scores than the control group. There was no significant difference in the scores of the other sub-parameters sensory search (*p* = .338), gustatory olfactory processing (*p* = .126) and visual processing (*p* = .110) in both groups (Table 3).

**Table 3:** Comparison of Computerized of Sensory Organization Test and Sensory Processing between groups

		Control Group Mean(IQR) X ± SD	CI Group Mean(IQR) X ± SD	*p*
**SOT**	SOM (%)	99,20 (96,00-103,00)	97,25 (75,00-102,00)	0.203
	VIS (%)	94.42 ± 4.59	72.21 ± 24,77	<,001**
	VEST (%)	81.74 ± 7.80 (,0–65,00)	32.16 ± 34.61 (43–82,00)	<,001**
	PREF (%)	83,19 ± 20,21	88,81 ± 16,80	,846
	Composite Score (%)	85,11 ± 3.87	60.47 ± 17.48	,001*
**Sensory** **Processing**	Low Registration	31.11 ± 3.28	39.63 ± 8.62	<,001**
	Sensation Seeking	44.84 ± 5.14	46.42 ± 7.22	0.338
	Sensory Sensitivity	35.95 ± 7.31	42.21 ± 11.37	0.014*
	Sensation Avoiding	35.89 ± 7.31	42.00 ± 12.03	0.035*
	Taste/Smell Processing	23.74 ± 3.73	23.26 ± 5.17	0.126
	Movement Processing	18.79 ± 3.13	21.79 ± 5.72	0.021*
	Visual Processing	27.26 ± 5.31	29.68 ± 7.17	0.110
	Touch Processing	33.53 ± 4.47	33.79 ± 8.87	0.002*
	Activity Level	27.00 ± 4.16	29.89 ± 7.71	0.006*
	Auditory Processing	29.89 ± 7.17	37.32 ± 6.16	0.024*

SOT: Sensory Organization Test, SOM: Somatosensory, VIS: Visiual, VEST: Vestibular, PREF. X ± SD: Mean ± Standart Deviation, Paired samples T Test *p < 0,05, Wilcoxon signed rank test *p < 0,05**p < 0,001

## Discussion

Sensory processing skills play a role in how infants and children learn about the world and how adults develop adaptive behavior. The cornerstone of behavior is the organization and integration of sensations from our environment and body [18]. Individuals’ sensory processing skills and current sensory profiles are very important in communication skills and in planning personalized treatment.

Sensorineural hearing loss due to different causes and degenerative processes occurring in the brain and sensory systems affect the ability to process and/or respond to sensory information [19, 20]. Auditory processing problems and weakening of the vestibular and proprioceptive systems are among the effects of this process. In addition, balance problems experienced after cochlear implant surgery may cause individuals to move more slowly, orientation problems and disorganization [21]. In terms of embryological development, sensorineural hearing loss (SNHL) is associated with vestibular dysfunction, which is one of the causes of balance disorders [22]. The occurrence of vestibular problems following Cl surgery to restore hearing ability in patients with SNHL is thought to be related to surgically induced vestibular dysfunction [23].

We used the Computerised Dynamic Posturography (CDP) to assess balance function in CI users, evaluating the Sensory Organisation Test (SOT) analysis. The SOT analysis of the CDP is a device that provides a good quantitative measure to determine the type of sensory abnormality responsible for balance impairment and provides detailed information about balance by analyzing the visual, proprioceptive and vestibular information of the three sensory systems affecting balance, the motor system, the central responses of the lower limbs and body movements assessed in different situations. This is important for selecting the treatment strategy as well as for monitoring the improvement in the patient’s condition during treatment [24]. In our study, we obtained vestibular, visual and compsite scores below the normal limit in CI users, and we also obtained worse results and a significant difference compared to participants with normal hearing, which showed us that CI users had poorer balance functions than normal participants. We investigated the link between this and sensory processing skills.

Among the groups, individuals using CI implants scored higher in the low register step. The fact that low register values are different from normal indicates that individuals may have problems in responding to stimuli [17, 20]. The difficulty increases especially if there are weak or less obvious auditory stimuli. They may miss clues in the environment, they may not be able to distinguish auditory stimuli, and they may have difficulty in balance and coordination while walking or during physical activities by not noticing changes in their surroundings [20]. We thought that the low auditory stimulus and high recording score findings in individuals with CI may cause auditory perception and vestibular discrimination difficulties.

In our study, it was shown that CI users have sensitivities in activity level and movement processing skills. They were more prone to sensory avoidance, in line with the literature [1]. Studies show that cochlear implant surgeries affect the vestibular system [21, 25–27]. Our CDP results, which are used to evaluate the type or severity of vestibular impairment, clearly revealed vestibular loss. In particular, vestibular, visual and composite scores, which are sub-parameters of the CDP, were below the normal limit and we obtained a significant difference when compared with normal age groups. We think that the result of the study that cochlear implant users are prone to sensory avoidance may be due to the fact that the individual moves slower and more cautiously to protect himself due to the vestibular sensitivity that emerged after surgery. After surgery, the vestibular system is expected to improve compensatoryly with the start of implant use and functional daily life. However, in our study, it was observed that individuals had balance problems for a long time. Restricted movement causes less stimulus input to the vestibular and proprioceptive systems. This vicious circle and difficulties in auditory perception and auditory discrimination skills may lead to social isolation and difficulties in daily living skills. The decrease in individuality and independence increases the risk of anxiety and depression [28]. Emotional regulation may create differences in sensory processing skills in individuals [29, 30]. In our study, the difference between the quality of life of the two groups was found to be significant in emotional well-being and social function parameters (*p* < .05).

The sensory profiles of individuals using CI are important in terms of communication skills, improving quality of life, and implementing personalized intervention programs for planned auditory rehabilitation.

## Conclusion

We believe that sensory disturbances, sensory dysregulation and vestibular dysfunction should not be overlooked in individuals with Cl. Whenever possible, all patients requiring cochlear implant surgery should be screened for vestibular evaluation and sensory profile, and postoperative evaluation should also be performed. We believe that individualized rehabilitation planning is necessary for disabilities resulting from hearing loss. Our findings revealed the necessity of multisensory assessment and therapy when working with individuals using cochlear implants.
